# An Anti-Vivisection Manifesto

**Published:** 1909-02-06

**Authors:** Sidney Trist

**Affiliations:** (Secretary.) London and Provincial Anti-Vivisection Society, Regent Street, S.W.


### EDITOR'S LETTER-BOX.

### AN ANTI-VIVISECTION MANIFESTO.

Sir,—When Dr. Emanuel Klein gave very frank but somewhat unpleasing evidence from the standpoint of his fellow-physiologists at the Royal Commission of 1877, Professor Huxley, who was a member of the Commission, and in favour of the practice of vivisection, wrote roundly denouncing Dr. Klein; it was held that he was not a representative physiologist. Why should you regard, therefore, Mrs. Cowan, the writer of the manifesto to which you and most anti-vivisectionists object, as representing the whole anti-vivisection party? I have already written to the *Times* and other newspapers saying how we deplore that circular, which we were powerless to prevent the circulation of because an individual has his or her own rights in a free country; and it were better that the right of freedom of expression should be safeguarded than that there should be no freedom of expression at all. But that does not prevent me or any other anti-vivisectionist from dissociating himself or herself from Mrs. Cowan's well-meant but distasteful method of fighting vivisection. When you write as if the whole anti-vivisection position was absurd, you appear to forget that in so doing you are roundly denouncing some of the greatest men and women—not from the social standpoint, but from that of mental and moral personality—which the last century has produced. In denouncing over-zeal and fanaticism, I think we may fairly expect from a journal which claims to be calmly scientific, that it shall preserve a judicial temper and not confuse one person with the whole party, nor attempt to fix on them the indiscretion of a single person.

I am, sir, your obedient servant,

Sidney Trist (Secretary.)

London and Provincial Anti-Vivisection Society,
Regent Street, S.W.

January 27, 1909.

[We are very glad to publish this disclaimer from Mr. Trist, and we do not doubt that he regards Mrs. Cowan's manifesto, notwithstanding his qualifying adjective of "well-meant," as distinctly immoral, just as we do. We take leave to doubt, however, whether that lady is as unsupported in the anti-vivisectionist party as Mr. Trist hopes she is.—Ed. T. H.]

